# Probing fMRI brain connectivity and activity changes during emotion regulation by EEG neurofeedback

**DOI:** 10.3389/fnhum.2022.988890

**Published:** 2023-01-06

**Authors:** Amin Dehghani, Hamid Soltanian-Zadeh, Gholam-Ali Hossein-Zadeh

**Affiliations:** ^1^School of Electrical and Computer Engineering, University of Tehran, Tehran, Iran; ^2^Department of Psychological and Brain Sciences, Dartmouth College, Hanover, NH, United States; ^3^Medical Image Analysis Lab, Department of Radiology and Research Administration, Henry Ford Health System, Detroit, MI, United States; ^4^School of Cognitive Sciences, Institute for Research in Fundamental Sciences (IPM), Tehran, Iran

**Keywords:** neurofeedback, simultaneous recording of EEG and fMRI, emotion regulation, frontal asymmetry, functional connectivity, autobiographical memory

## Abstract

Despite the existence of several emotion regulation studies using neurofeedback, interactions among a small number of regions were evaluated, and therefore, further investigation is needed to understand the interactions of the brain regions involved in emotion regulation. We implemented electroencephalography (EEG) neurofeedback with simultaneous functional magnetic resonance imaging (fMRI) using a modified happiness-inducing task through autobiographical memories to upregulate positive emotion. Then, an explorative analysis of whole brain regions was done to understand the effect of neurofeedback on brain activity and the interaction of whole brain regions involved in emotion regulation. The participants in the control and experimental groups were asked to do emotion regulation while viewing positive images of autobiographical memories and getting sham or real (based on alpha asymmetry) EEG neurofeedback, respectively. The proposed multimodal approach quantified the effects of EEG neurofeedback in changing EEG alpha power, fMRI blood oxygenation level-dependent (BOLD) activity of prefrontal, occipital, parietal, and limbic regions (up to 1.9% increase), and functional connectivity in/between prefrontal, parietal, limbic system, and insula in the experimental group. New connectivity links were identified by comparing the brain functional connectivity between experimental conditions (Upregulation and View blocks) and also by comparing the brain connectivity of the experimental and control groups. Psychometric assessments confirmed significant changes in positive and negative mood states in the experimental group by neurofeedback. Based on the exploratory analysis of activity and connectivity among all brain regions involved in emotion regions, we found significant BOLD and functional connectivity increases due to EEG neurofeedback in the experimental group, but no learning effect was observed in the control group. The results reveal several new connections among brain regions as a result of EEG neurofeedback which can be justified according to emotion regulation models and the role of those regions in emotion regulation and recalling positive autobiographical memories.

## Introduction

Emotion regulation consists of control and management of emotional states, mood, and affect with specific strategies, e.g., situation selection, situation modification, attentional deployment (e.g., distraction and rumination), cognitive change (e.g., reappraisal and distancing), and response modulation (e.g., exercise) (Gross, 1998; Koole, 2009; Aldao, 2012). The common strategies for emotion regulation can be categorized into implicit and explicit strategies based on conscious awareness of the goal of the regulation strategy which can be explained to participants or patients (Balconi et al., 2018).

Neurofeedback is a non-invasive brain training procedure with various clinical and non-clinical applications especially for emotion regulation (Sitaram et al., 2017). In neurofeedback, feedback is provided to a subject based on his/her brain activity in order to self-regulate his/her brain function.

Electroencephalography and fMRI are the two main neuroimaging modalities used in the study of neurofeedback for emotion regulation. Simultaneous recording of EEG and fMRI provides complementary information and allows for a more comprehensive understanding and research in neurofeedback by exploring EEG and fMRI correlation (EEG-informed fMRI), fusion analysis, and validation of the effectiveness of the applied paradigm for a specific purpose (Ebrahimzadeh et al., 2019a,b, 2021, 2022; Mosayebi et al., 2022). EEG-based neurofeedback has applications in the treatment of attention-deficit/hyperactivity disorder (ADHD) (Lubar et al., 1995; Zuberer et al., 2018), schizophrenia (Bolea, 2010), insomnia (Hammer et al., 2011), drug addiction (Lackner et al., 2016), autism (Coben et al., 2010), epilepsy (Saxby and Peniston, 1995; Walker and Kozlowski, 2005; Kaur and Singh, 2017; Linhartová et al., 2019), anxiety (Mennella et al., 2017), pain (Kubik and Biedroń, 2013, eating disorders (Bartholdy et al., 2013), Parkinson disease (Rossi-Izquierdo et al., 2013), obsessive-compulsive disorder (Hammond, 2003), post-traumatic stress disorder (PTSD) (Gapen et al., 2016) in addition to other psychological applications e.g., emotion regulation (Dennis and Solomon, 2010; Quaedflieg et al., 2015; Linhartová et al., 2019).

Most of the EEG neurofeedback protocols try to modulate the EEG signal amplitude or power in particular frequency bands (Sourina et al., 2012; Peeters et al., 2014b; Allen and Reznik, 2015; Kelley et al., 2017; Mennella et al., 2017; Meyer et al., 2018). Recent development in MRI pulse sequences has made it possible to use fMRI for neurofeedback which is named time-real fMRI (Subramanian et al., 2011; Koush et al., 2013; Sulzer et al., 2013; DeBettencourt et al., 2015; Kim et al., 2015; Megumi et al., 2015; Sarkheil et al., 2015; Cohen Kadosh et al., 2016; Sherwood et al., 2016). EEG neurofeedback with simultaneous fMRI has been proposed in previous studies (Zotev et al., 2016, 2020; Lioi et al., 2020a; Perronnet et al., 2020), Theta/Alpha power ratio was used by Kinreich et al. (2012) to enhance relaxation in subjects, which is useful in a range of clinical applications such as PTSD and ADHD.

EEG frontal asymmetry has been used in several studies for emotion regulation based on the approach – withdrawal model proposed by Davidson et al. (1990). According to this model, withdrawal emotion or negative affect (such as fear, sadness, and disgust) is associated with higher activity in the right hemisphere. Conversely, increasing activity in the left hemisphere is associated with emotional states such as joy or anger (Davidson et al., 1990; Quaedflieg et al., 2015). Several applications of EEG frontal asymmetry are listed in Coan and Allen (2004). As mentioned in several previous studies (Coan and Allen, 2004; Peeters et al., 2014a; Allen and Reznik, 2015; Mennella et al., 2017; Allen et al., 2018; Meyer et al., 2018; Xu et al., 2018; López-Castro et al., 2021; Trambaiolli et al., 2021; Weon et al., 2021), EEG frontal asymmetry can be used as an indication of different emotional states and a biomarker for PTSD, anxiety, and depression.

In the study by Peeters et al. (2014b), the EEG frontal asymmetry was used for both up/down emotion regulation in a single session of neurofeedback training. In this study, it was hypothesized that positive/negative affect would result in increased activity of the left/right frontal hemisphere and decreased activity of the right/left frontal hemisphere. The results revealed that participants were able to up/down-regulate their EEG frontal asymmetry using neurofeedback. Similar to most previous neurofeedback studies (Marxen et al., 2016; Wang et al., 2016; Vourvopoulos et al., 2019; Klöbl et al., 2020; Lioi et al., 2020a), this study lacked a control group for receiving sham neurofeedback.

Simultaneous recording of EEG and fMRI during emotion regulation was used in several previous studies, especially when using EEG frontal asymmetry alone or both EEG frontal asymmetry and fMRI as neurofeedback. In Cavazza et al. (2014), EEG neurofeedback based on frontal alpha asymmetry with simultaneous fMRI was used based on interactive narrative paradigms. The success rate of emotion regulation and changing the frontal asymmetry in this study was low. In addition, this study did not include a control group.

In the studies by Zotev et al. (2014, 2018b), emotion regulation was done through retrieving positive autobiographical memories. The neurofeedback in these studies was based on both EEG frontal asymmetry and BOLD signal of the amygdala. The results demonstrated the effectiveness of simultaneous EEG-fMRI neurofeedback for emotion regulation but the redundancy of neurofeedback based on both modalities was not clarified in these studies.

According to a recently published review paper in emotion regulation using fMRI neurofeedback (Linhartová et al., 2019), several brain regions including the amygdala, insula, anterior cingulate cortex (ACC), and prefrontal/frontal regions (dorsomedial prefrontal cortex (DMPFC), dorsolateral prefrontal cortex (DLPFC), and orbitofrontal cortex (OFC)) are involved in emotion regulation and treatment of mental disorders such as depression, PTSD, and anxiety. In some previous studies (Zotev et al., 2011, 2013, 2018b; Koush et al., 2017; Young et al., 2018), interactions among a small number of regions e.g., the amygdala and other regions were evaluated. Notwithstanding several existing emotion regulation studies using neurofeedback or retrieval of autobiographical memories, understanding the interaction of the brain regions involved in emotion regulation through neurofeedback still needs further investigation by considering brain regions with key roles in emotion regulation. Therefore, we implement simultaneous fMRI–EEG recording during an EEG neurofeedback using an induced implicit happiness task for emotion regulation through positive autobiographical memories. Initially, we wanted to use both EEG and fMRI for neurofeedback, but because of technical problems, we used EEG as neurofeedback.

Therefore, this study aims to investigate how EEG neurofeedback changes EEG and fMRI BOLD signals, psychometric assessments, and connectivity of all activated brain regions during emotion regulation and separate the effect of paradigm and neurofeedback during emotion regulation. Using different approach from our previous study (Dehghani et al., 2020, 2021, 2022), we use general GLM as a model-based method to extract activated brain voxels/regions during emotion regulation. We test the hypothesis that EEG frontal asymmetry, activity and the connectivity in/between the prefrontal, limbic, and insular regions change through EEG neurofeedback. The connectivity results can be used for the design of new neurofeedback paradigms for emotion regulation, especially using real-time fMRI, as a therapeutic tool for the treatment of mental disorders.

## Materials and methods

### Task design

The research protocol is approved by the ethics committees of the Iran University of Medical Sciences, Tehran, Iran. 20 healthy subjects (age 26.7 ± 3.6 years, all male) as the experimental group, and 15 healthy subjects (age 27 ± 3.8 years, all male) as the control group participated in this study. Participants in the control group are provided (without their knowledge) with sham EEG neurofeedback. We had no drop out according to quality control of data, but we had three drop out before starting the experiments because of technical problems (two subjects of experimental group and one subject of control group). The exclusion criteria are a prior history of major psychiatric or neurological disorder, drug or alcohol abuse during the past year, brain surgery, and contraindications to MRI and all participants were right-handed with the normal or corrected-to-normal vision. Before the experiment, two psychometric tests including Beck’s Depression Inventory (Craven et al., 1989) and General Health Questionnaire – 28 (GHQ-28) (Nazifi et al., 2014) are completed by each participant. The mean ± standard deviations of Beck’s Depression Inventory and GHQ-28 for all participants are 6.8 ± 3 and 2.2 ± 2, respectively. Therefore, the participants are normal and non-psychiatric according to the scores of the Beck’s Depression Inventory (all participants have scores less than or equal to 12 and are classified as normal or minimum depression, 24 participants have scores less than 9) (Khademi et al., 2005; Smarr and Keefer, 2011) and GHQ-28 tests (all participants have scores less than or equal to 7 and classified as non-psychiatric, 28 participants have scores less than or equal to 3) (Goldberg et al., 1998; Hjelle et al., 2019). All participants are examined by a resident physician before the experiments (to check their blood pressure and inform them about the experimental environment) and fill out the consent form for participation in the experiments.

**FIGURE 1:**
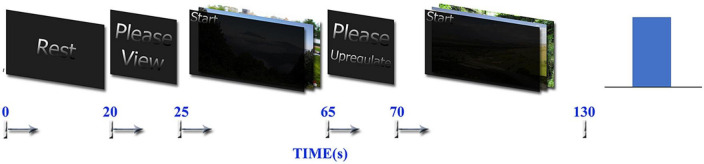
Neurofeedback bar and timing of the tasks used in the one run of neurofeedback protocol, which contains Rest, View, and Upregulation blocks.

Retrieving positive autobiographical memories is a common strategy used for upregulating positive emotions (Linhartová et al., 2019). The experimental paradigm in this study is similar to the one published in Young et al. (2014), Zotev et al., 2014, 2016, 2018a but with minor differences and is based on retrieving autobiographical happy memories. Before the experiment, each participant was interviewed and asked to write several positive autobiographical memories. The experiment contains 10 runs, and each participant has 10 blocks of emotion regulation using various autobiographical memory retrievals. The duration of each run is 130 s and total duration of whole experiment is 1300 s. Each run contains three blocks including Rest, View, and Upregulation. The difference between our paradigm and similar paradigms (Young et al., 2014; Zotev et al., 2014, 2016) is that we select pictures based on what subjects announce during interviews and present them during the experiments to remind the autobiographical memories related to these pictures in each run. The duration of the Rest block is 20 s, the View block is 40 s, and the Upregulation block is 60 s. During the Rest block, no image is shown, and only the message “please rest” is displayed on the screen to ask the participants to relax with no specific tasks. In the View block, two related pictures were presented for the 40s and the participant is asked to see them without thinking about them or remembering anything. In the Upregulation block, two images similar to the View block are presented and the participant tries to increase the height of the bar of neurofeedback based on the brain activity. The selected images for View and Upregulation blocks have similar arousal and valence (without any significant difference) according to the rates given after the experiment by each participant to individual images of positive autobiographical memories. The whole neurofeedback protocol for one run is depicted in Figure 1.

Before the experiment, we explain the paradigm to each participant by the visual presentation of a sample run of the task. Then, we ask them to try to increase the height of the bar of the neurofeedback during the Upregulation block by remembering the positive autobiographical memories (without showing the individual pictures). In each step, the bar is blue if the participant succeeds to increase or maintain the brain activity represented by neurofeedback and red if he does not succeed.

The neurofeedback in the experimental group is based on the approach–withdrawal hypothesis (Davidson et al., 1990), which is defined as the difference between the EEG power in the right and left hemispheres in the alpha frequency band and in the windows with a length of 2 s, which is updated every 1 s with 50% overlap between the consecutive windows (the height of the neurofeedback bar at each time is the average of the EEG asymmetry alpha power in the current and two previous windows). For the control group, the sham feedback is a randomly generated signal for each Upregulation block as proposed in Zotev et al. (2018a). Neurofeedback is presented only in the Upregulation block.

### Data acquisition

The MRI data are acquired using a 3.0 Tesla Siemens Prisma MRI Scanner located in the National Brain Mapping Lab (NBML), Tehran, Iran. Functional MRI are acquired using a T2*-weighted gradient-echo, echo-planar (EPI) pulse sequence (TR = 2000 ms, TE = 30 ms, flip angle = 90°, matrix size = 64 × 64 × 30, and voxel size = 3.8 × 3.8 × 4 mm). During the 10 runs of the experiment session, 650 volume images (1300 s) are acquired. Structural images are acquired using a gradient-echo, T1-weighted MPRAGE pulse sequence (TI = 1100 ms, TR = 1810 ms, TE = 3.47 ms, and voxel size = 1 × 1 × 1 mm).

The EEG data are recorded simultaneously with fMRI using the Brain Products EEG system. The EEG cap has 64 electrodes (FCz as the reference and AFz as the ground electrode) according to the 10–20 system and one ECG electrode. Electrode impedances are maintained below 5K Ohms by cleaning the skin and removing dirt using alcohol and injecting suitable gel. The EEG signal is recorded at 5K samples/sec. For quality control of the EEG data and neurofeedback, several minutes of the recording are performed in the MRI control room just before the subject goes in the MRI scanner. The task is presented by the Psychtoolbox program through a coil mounted display, which allows the subjects to see the stimulus and different blocks of the experiment.

### Real-time data processing

Due to practical limitations, neurofeedback is provided only based on the EEG signal. For the EEG neurofeedback, the RecView software is applied to remove MRI and ballistocardiogram artifacts from the EEG data in real time, using the moving average method (Allen et al., 1998, 2000). As the mean head displacement obtained in offline analysis is 0.41 ± 0.17 mm (in the range of [0.10 mm 0.90 mm], all participants except for one had head motion less than 0.60 mm), the result of the moving average template subtraction method will not differ significantly from the counterpart methods (Niazy et al., 2005; Moosmann et al., 2009). In addition, since the head movement and ballistocardiogram artifacts induce large peaks in the EEG signal, the remaining large peaks are excluded. Removing the ballistocardiogram residual from the EEG signal is very difficult. However, because of small head displacements and since the frequency range of this artifact is less than 7 Hz, our feedback which is in the alpha frequency band will not be affected by the artifact.

The denoised data is down-sampled to 250 samples/sec. Then, the powers of channels F3 and F4 are calculated every 1 s using a 2-s moving window. The relative EEG power asymmetry for F4 and F3 with respect to the baseline (by averaging the asymmetry values in the previous View block) is calculated and presented as a bar during Upregulation blocks ((Ln(F4)−Ln(F3))_in each 2sec window_−(Ln(F4)−Ln(F3))_previous View block_). For high-quality neurofeedback, the following steps are done. First, the power spectrum of the data recorded inside and outside of the MRI scanner is compared to evaluate the quality of the denoised data. Secondly, the spectrum of neurofeedback and its values obtained by RecView are compared with the results of the offline analysis to evaluate the neurofeedback quality. The results confirm the quality of the offline removal of the EEG artifacts and the reliability of the neurofeedback. They also prove that the neurofeedback provided to each participant is based on the actual brain activity, not the artifacts.

### EEG data analysis

An offline analysis of the EEG data acquired simultaneously with fMRI, is performed using the FMRIB plug-in as a Matlab toolbox (Christov, 2004; Kim et al., 2004; Niazy et al., 2005). This software removes the fMRI gradient artifacts, detects the QRS complexes from an ECG channel, and removes the pulse (ballistocardiography/BCG) artifacts from the EEG signal. The MR artifact removal method includes 4 steps. In the first step, slice-timing triggers are adjusted to optimize the alignment of triggers. Second, a local artifact template subtraction method is performed. Third, the artifact residuals are estimated using basis functions derived from performing PCA on each channel’s artifact segments. The moving average subtraction performed in stage 2 is adaptive and removes more than 98% of the artifacts and PCA removes the residuals. Fourth, adaptive noise cancellation (ANC) is used. ANC removes any components in the data that are correlated with a reference. By using the subtracted noise as a reference (from the second and third step) artifact components not captured in the basis set are removed. More details are described in Niazy et al. (2005). For the BCG, a robust QRS complex detection algorithm is used (Niazy et al., 2005). Then, optimal basis sets method used in the third step of MR artifact removal is used to remove BCG artifact. The validation of these methods was demonstrated in Niazy et al. (2005). After removing the MRI and BCG artifacts, the EEG data are down-sampled to 250 samples/sec and low-pass filtered at 100 Hz. Next, the fMRI slice selection frequency and its harmonics are removed by band-stop filtering (15, 30, 45, 60, 75, and 90 Hz). Then, ICA is applied over the entire EEG data after excluding the noisy and motion-affected intervals. Next, independent components (ICs) corresponding to the artifacts, e.g., eye blinking, head movement, neck, head, and shoulder which included Scalp/face and muscle EMG, and cardioballistic or BCG residual, are identified and removed. This is done based on the time course spectral density (head movement in the range of 0.5–4.5 Hz, cardioballistic motion in the range of 2–7 Hz, eye blinking in the range of 0.5–3 Hz), topographic map (bipolar topography for BCG) (Mayeli et al., 2016), and kurtosis (rapid and random head movements have high kurtosis values) (Mognon et al., 2011; Zotev et al., 2012; Wong et al., 2016). Then, the average EEG power spectrum is calculated for each of the experimental blocks (Upregulation, View, and Rest). A moving window with a length of 2 s and 50% interval overlap is applied to the EEG data to calculate the EEG asymmetry of channels F3 and F4 in the alpha band for each block. To evaluate the effect of heart rate variability (HRV) on emotion regulation, HRV was extracted for all participants in three blocks of the experiment, using their ECG channel recorded during the experiment.

### fMRI data analysis

An offline analysis of the whole brain fMRI data is performed in FSL. Pre-processing of a single-subject fMRI data includes slice-timing correction, motion correction, temporal high pass filtering (cut-off = 0.005 Hz), and spatially smoothing using an 8 mm full-width at half-maximum Gaussian kernel. The standard GLM analysis is then applied to the fMRI time series. Three regressors for Upregulation, View, and Rest are convolved with the hemodynamic response function and six motion confounds are included in the GLM model. Finally, the whole brain is thresholded at *p*-value = 0.01 for voxels and for cluster correction at *p*-value = 0.01 in the cluster-level correction algorithm, which corrects for the multiple comparisons using the Gaussian Random Field (GRF) model (Smith et al., 2004; Fsl, 2006; Woolrich et al., 2009). To determine the active regions in the Upregulation blocks, the contrasts of the “Upregulation versus View” and the “Upregulation versus Rest” are calculated. A global measure of the signal change ($\frac{\text{mean}\,\left(\text{Upregualtion}\right)-\mathrm{mean}\,\left(\mathrm{View}\right)}{\mathrm{mean}\,\left(\mathrm{View}\right)}$ or $\frac{\text{mean}\,\left(\text{Upregualtion}\right)-\mathrm{mean}\,\left(\mathrm{Rest}\right)}{\mathrm{mean}\,\left(\mathrm{Rest}\right)}$) in different activated regions of the preprocessed fMRI data registered to the Montreal Neurological Institute (MNI) atlas is calculated using an anatomical mask. For this purpose, anatomical masks from “WFU_PickAtlas” and FSL were used to label the activation clusters. Then, the mean signal changes of the activated voxels in every active region for Upregulation versus View and Rest are calculated in the 118 anatomical masks extracted from the “WFU_PickAtlas” and FSL (Tzourio-Mazoyer et al., 2002; Maldjian et al., 2003, 2004; Desikan et al., 2006; Gorgolewski et al., 2015). WFU_PickAtlas provides a method for generating ROI masks based on the Talairach Daemon database. The atlases include Brodmann area, Lobar, Hemisphere, Anatomic Label and Tissue Type. WFU_PichAtlas has totally 116 regions and we used them to find the activated brain regions.

For further evaluation, Pearson’s correlation is used to estimate and compare the functional connectivity in the different blocks of the experiment. To this end, the anatomical masks of various brain regions are used to extract the mean BOLD signals of the activated voxels in the regions and calculate the functional connectivity between the regions. The results are used to reveal the neural mechanisms of the neurofeedback protocol (functional connectivity) in the brain. To remove the effect of the hemodynamic response of the View block on the Upregulation block, the first 4 s (two samples) of the BOLD signal of each Upregulation block is removed for signal change calculation and functional connectivity analysis.

### Psychometric testing

To measure changes in the mood state, each participant completes the Persian version of Beck Anxiety Inventory (BAI), State-Trait Anxiety Inventory (STAI), Positive-Negative affect scale (PANAS), and short Persian version of the Profile of Mood States (POMS) before and after the neurofeedback test. The Beck Anxiety Inventory (BAI) contains 21 questions and measures the severity of anxiety (Beck et al., 1988). The State-Trait Anxiety Inventory (STAI) includes 20 items for state anxiety and 20 items for trait anxiety (Azizi et al., 2013). The POMS includes a self-rating of six different aspects of the mood, e.g., Tension or Anxiety, Anger or Hostility, Vigor or Activity, Fatigue or Inertia, Depression or Dejection, and Confusion or Bewilderment, with a five-point scale rating from “not at all” to “extremely” (Spielberger, 1972). The PANAS also contains a self-rating of the positive and negative affects with a five-point scale rating from “not at all” to “very much” (Watson et al., 1988).

## Results

### EEG results

**FIGURE 2:**
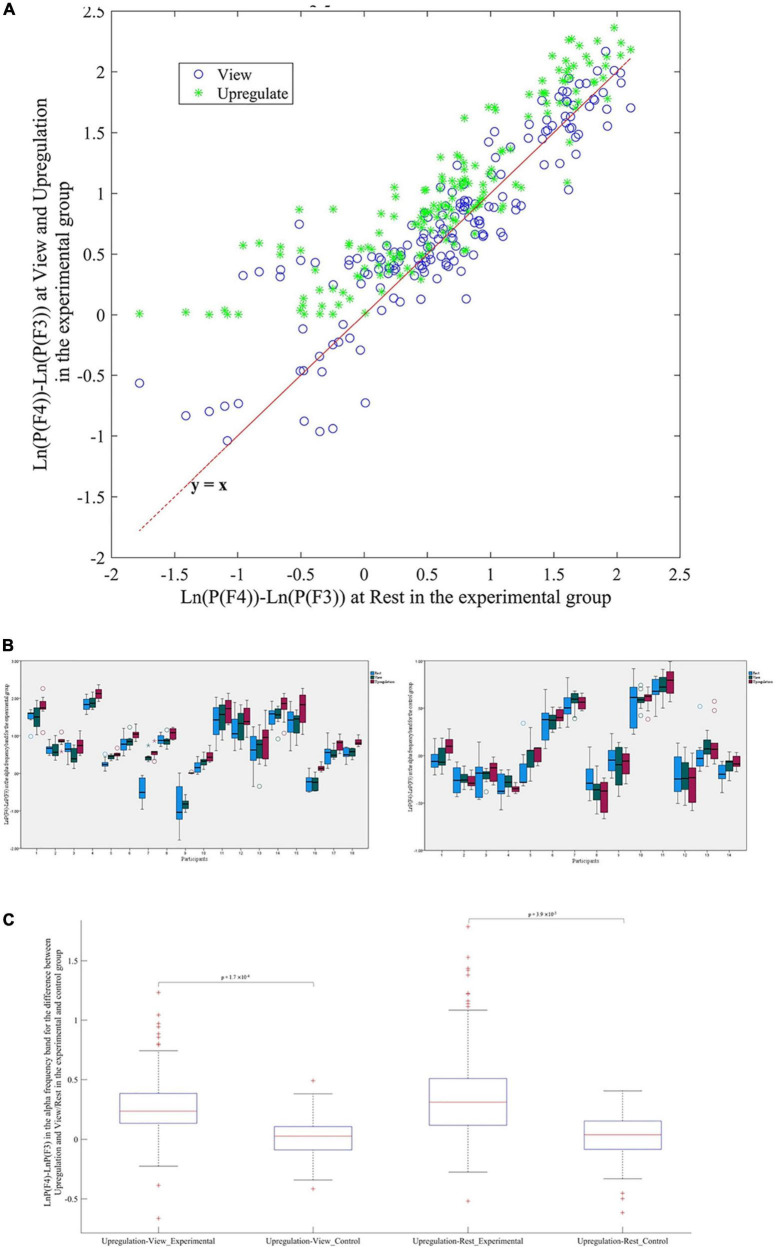
**(A)** Scatter plot of frontal asymmetry in blocks of View and Upregulation versus Rest for the experimental group. **(B)** Boxplot of frontal asymmetry in blocks of Rest, View, and Upregulation for the experimental and control groups. **(C)** Boxplot of frontal asymmetry for Upregulation versus View and Rest in the experimental and control groups.

The EEG power in the alpha band is used for neurofeedback. The average change of the alpha power asymmetry (LnP(F4)-LnP(F3)) in the Upregulation, View, and Rest blocks for the experimental and control groups are illustrated in Figure 2. Since the feedback for the experimental group is based on the powers of channels F4 and F3, and the subjects try to increase LnP(F4)-LnP(F3) in the alpha frequency band versus the View period, we expect the mean power of “LnP(F4)-LnP(F3)” in the Upregulation block to be higher than the Rest and View blocks for the experimental group as illustrated in Figures 2A, B. The changes in the EEG asymmetry for the Upregulation versus View/Rest periods are significant for the experimental group (10 runs ×18 participants = 180 data for each Upregulation, View and Rest blocks of experimental group, using the “Kolmogorov–Smirnov test to confirm normality of data, Repeated Measures ANOVA with Upregulation, View, and Rest as independent variable, *F*(2,537) = 12.08, *p*-value < 0.001, Repeated Measures ANOVA with Upregulation and Rest as independent variable, *F*(1,358) = 21.85, *p*-value < 0.001, Repeated Measures ANOVA with Upregulation and View as independent variable, *F*(1,358) = 14.51, *p*-value < 0.001). Also, the changes in the EEG frontal asymmetry for the Upregulation versus View and Rest in the experimental group are significantly different from those in the control group (two sample *t*-test; *t*(318) = 3.62, *p*-value_Upregulation–View  (Experimental  group–Control group)_ = 1.7 ×10^–4^; and *t*(318) = 2.68; *p*-value_Upregulation–Rest (Experimental group–Control group)_ = 3.9 ×10^–3^, FDR-corrected for multiple comparison, *q* = 0.05).

Figures 2B, C show the effectiveness of the neurofeedback protocol as the difference between the frontal asymmetry of Upregulation and View/Rest in the experimental group are higher than in the control group. Consistent with the approach–withdrawal model that states during happiness, the difference between the power of the right and left hemispheres increases, the difference between the EEG power of channels F4 and F3 in the Upregulation blocks is higher than the View and Rest blocks.

To evaluate the effect of heart rate variability (HRV) on emotion regulation, HRV was extracted for all participants in three blocks of the experiment, using their ECG channel recorded during the experiment. The results of HRV analysis using the recorded ECG channel show that HRV in the Upregulation block is significantly different from those of the View and Rest blocks in only 3 of the 32 participants. This indicates that emotion regulation is not associated with HRV.

### fMRI results

Activation detection in the whole brain using GLM with different contrasts of Upregulation versus View and Rest illustrate several regions for the experimental group during emotion regulation. As mentioned earlier, to have a global evaluation, we use an anatomical mask for each region to compare the mean signal change of the activated regions between the Upregulation and Rest/View blocks. The results of significant signal changes for the Upregulation versus View and Rest (Repeated Measures ANOVA with Upregulation, View, and Rest as independent variable for every one of the 118 ROIs used in this study, FDR-corrected for multiple comparison; *q* = 0.05) and the corresponding *t*-value and Cohen’s D effect size for the experimental group are listed in Table 1.

**TABLE 1:** Percentages of signal change, t-score, and Cohen’s D effect size for brain regions with significant signal change between Upregulation versus View and Rest in the experimental group (FDR-corrected for multiple comparison, *q* = 0.05).

Regions	Sig % up-view	Sig % up-rest	t-score (up-view) – MNI coordinate	Cohen’s D effect size (up-view)	Sig % other researches (up-rest)
Left amygdala	0.86	0.70	4.9 (−24, 0, −12)	0.87	0.7 [74], 0.3, 0.1 [101], 0.2 [39]
Right amygdala	0.65	0.72	3.9 (24, 6, −16)	0.70	0.4 [74], 0.3 [44]
Left insula	1	0.64	7.7 (−44, 4, −4)	0.85	0.5 [99], 0.5 [101]
Right insula	0.91	0.62	6.4 (46, 6, 4)	0.88	–
Left anterior cingulate cortex	0.97	0.81	4.2 (−6, 12, 28)	0.95	0.3 [101]
Right anterior cingulate cortex	0.64	0.38	4.4 (18, 44, 14)	0.86	
Left cuneus	0.45	1.56	4.5 (−20, −56, 26)	0.61	0.5 [99]
Right cuneus	0.40	1.90	3.9 (26, −62, 26)	0.75	–
Left lingual gyrus	1.21	1.39	4.2 (−12, −68, −10)	0.83	–
Left posterior cingulate cortex	0.49	0.33	5 (−14, −52, 58)	0.66	0.5 [99]
Left thalamus	1.07	0.85	4.9 (−22, −24, 6)	0.93	–
Right thalamus	0.86	0.65	6.9 (18, −16, 18)	0.90	–
Left caudate	0.86	0.65	5.7 (−16, 12, 10)	0.80	–
Right caudate	0.74	0.49	8 (18, −14, 20)	0.80	–
Left hippocampus	0.57	0.56	4.3 (−36, −18, −12)	0.85	
Right hippocampus	0.44	0.59	4.2 (40, −26, −10)	0.80	–
Left dorsomedial prefrontal cortex	0.85	1.02	5.1 (0, 20, 42)	0.99	
Right dorsomedial prefrontal cortex	0.37	0.81	4.1 (10, 24, 42)	0.89	–
Left orbitofrontal cortex	1.13	1.04	6 (−44, 18, −2)	1	–
Right orbitofrontal cortex	1.12	0.81	6.5 (30, 32, −12)	0.85	–
Left middle temporal gyrus	0.66	0.59	5.9 (−48, −30, −26)	0.87	
Right middle temporal gyrus	0.69	0.70	5.9 (56, −20, −28)	0.70	–
Left ventral striatum	1.17	0.84	6.1 (−16, 19, −1)	1.03	0.5 [99]
Right ventral striatum	0.81	0.66	7.7 (19,16, −2)	0.65	0.5 [99]
Left ventrolateral prefrontal cortex	0.67	0.81	5.6 (−46, 18, 2)	0.82	0.5 [99]
Right ventrolateral prefrontal cortex	0.65	0.58	9.3 (62, 20, 0)	0.60	–
Left dorsolateral prefrontal cortex	0.84	0.90	5.3 (−28, 44, 18)	0.97	–
Right dorsolateral prefrontal cortex	0.76	0.75	6.7 (40, −2, 52)	0.88	–
Left superior parietal	0.46	0.69	4.9 (−24, −52, 50)	0.81	–
Right superior parietal	0.33	0.89	5.3 (16, −48, 54)	0.57	–
Left inferior parietal	0.60	0.41	5.9 (−60, −36, 44)	0.85	–
Right inferior parietal	1.32	0.53	4.2 (56, −36, 54)	1.01	–
Left supramarginal	0.87	0.42	8.1 (−54, −38, 24)	1.04	–
Left postcentral	0.67	0.56	6.8 (−50, −24, 32)	0.88	–

They illustrate several brain regions with large signal change and effect size for emotion regulation, which is consistent with the literature (listed in the last column of Table 1). The changes in the BOLD signal of brain regions in Table 1 for the Upregulation versus View and Rest in the experimental group are significantly different from those of the control group (two-sample *t*-test; df = 318; FDR-corrected for multiple comparison; *q* = 0.05).

**FIGURE 3:**
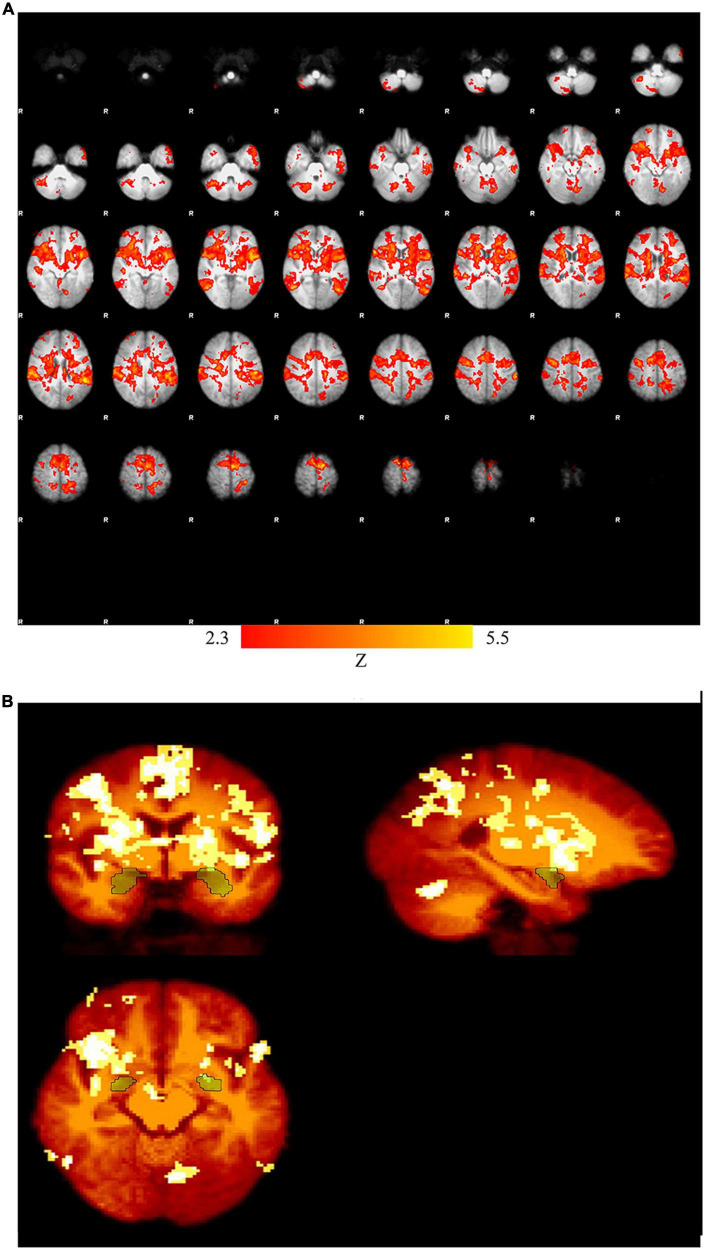
**(A)** Activation map for Upregulation versus View in the experimental group (p_corrected_ = 0.01), **(B)** activation map masked by amygdala.

Multiple brain regions especially in the subcortical regions play key roles in emotion regulation. The percentage of the signal change in the brain regions listed in Table 1 is higher than those of the previous studies. This may be due to the fact that the proposed emotion regulation paradigm (neurofeedback) is based on the recall of the positive autobiographical memory using the stimulus pictures related to the issues announced by the participants to induce happiness. However, other parameters e.g., protocol length and subject variability could be other sources of variability and higher signal changes. Figure 3 presents the activation map for the Upregulation versus View in the experimental group and the activation map masked by amygdala.

**FIGURE 4:**
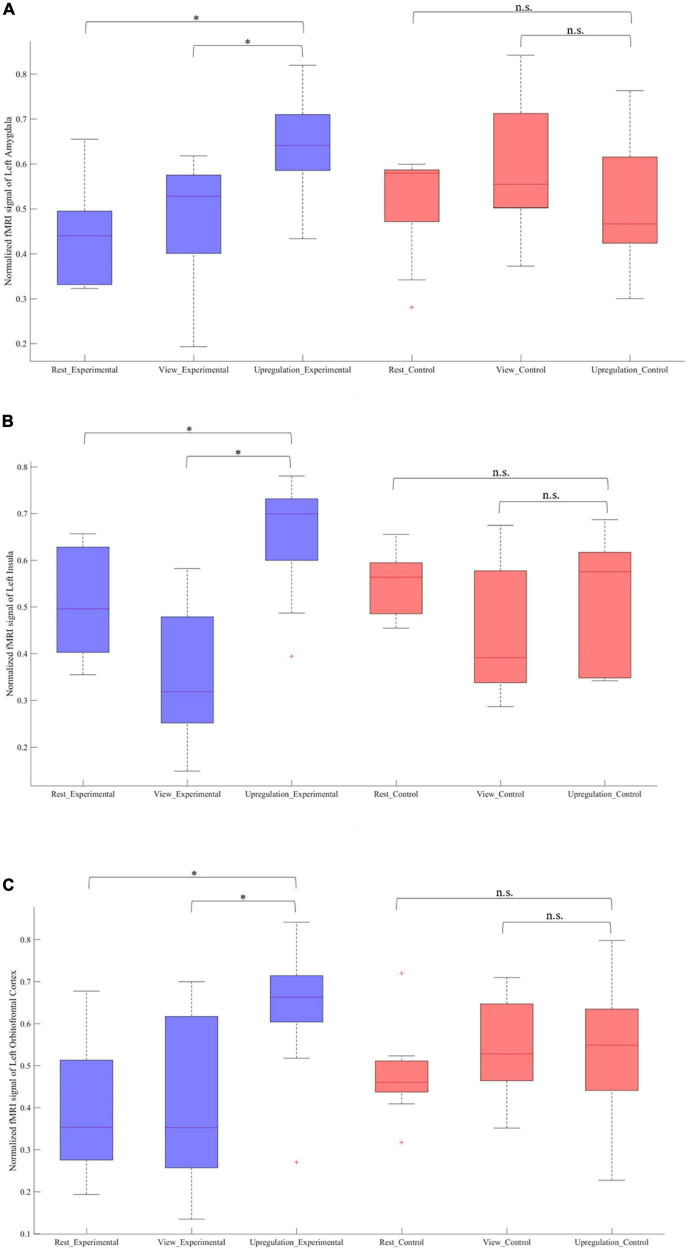
Normalized fMRI BOLD signal in the Rest, View, and Upregulation blocks for contrast of Upregulation versus View and Rest for the experimental and experimental groups in panel **(A)** left amygdala and *p*-value _Upregulation–View_ _(Experimental group–Control group) for left amygdala_ = 2.4 × 10^– 3^; *p*-value _Upregulation–Rest_ _(Experimental group–Control group) for left amygdala_ = 1.3 × 10^– 4^, **(B)** left insula and *p*-value _Upregulation–View_ _(Experimental group–Control group) for left Insula_ = 3.6 × 10^– 5^; *p*-value _Upregulation–Rest_ _(Experimental group–Control group) for left Insula_ = 5.9 × 10^– 4^, and **(C)** left orbitofrontal cortex and *p*-value _Upregulation–View_ _(Experimental group–Control group) for left OFC_ = 6.8 × 10^– 3^; *p*-value _Upregulation–Rest_ _(Experimental group–Control group) for left OFC_ = 1.5 × 10^– 5^ (*indicates *p* < 0.05 and n.s. indicates not significant).

Figure 4 provides the box plot of the signal changes in the most important regions for the Rest, View, and Upregulation states for all subjects in the experimental and control groups. In Figure 4, the signal change of each experimental/control group participant for each ROI is computed by subtracting a global minimum from the average intensity of that ROI and normalizing the result by the “difference between the global minimum and the global maximum.” Figure 4 shows higher signal changes for the Upregulation relative to the View and Rest for the experimental group. Also, these changes for the Upregulation versus View and Rest in the experimental group are significantly different from those in the control group (two sample *t*-test; *p*-value _Upregulation–View (Experimental group–Control group) for left amygdala_ = 2.4 × 10^–3^; *p*-value _Upregulation−Rest(Experimentalgroup−Controlgroup)forleftamygdala_ = 1.3 × 10^–4^, *p*-value _Upregulation−View (Experimentalgroup−Controlgroup) for left Insula_ = 3.6 × 10^–5^; *p*-value_Upregulation–Rest (Experimental group–Control group) for left Insula_ = 5.9 × 10^–4^, *p*-value _Upregulation–View (Experimental group–Control group) for left OFC_ = 6.8 × 10^–3^; *p*-value_Upregulation–Rest (Experimental group–Control group) for left OFC_ = 1.5 × 10^–5^; FDR-corrected for multiple comparison; *q* = 0.05).

**FIGURE 5:**
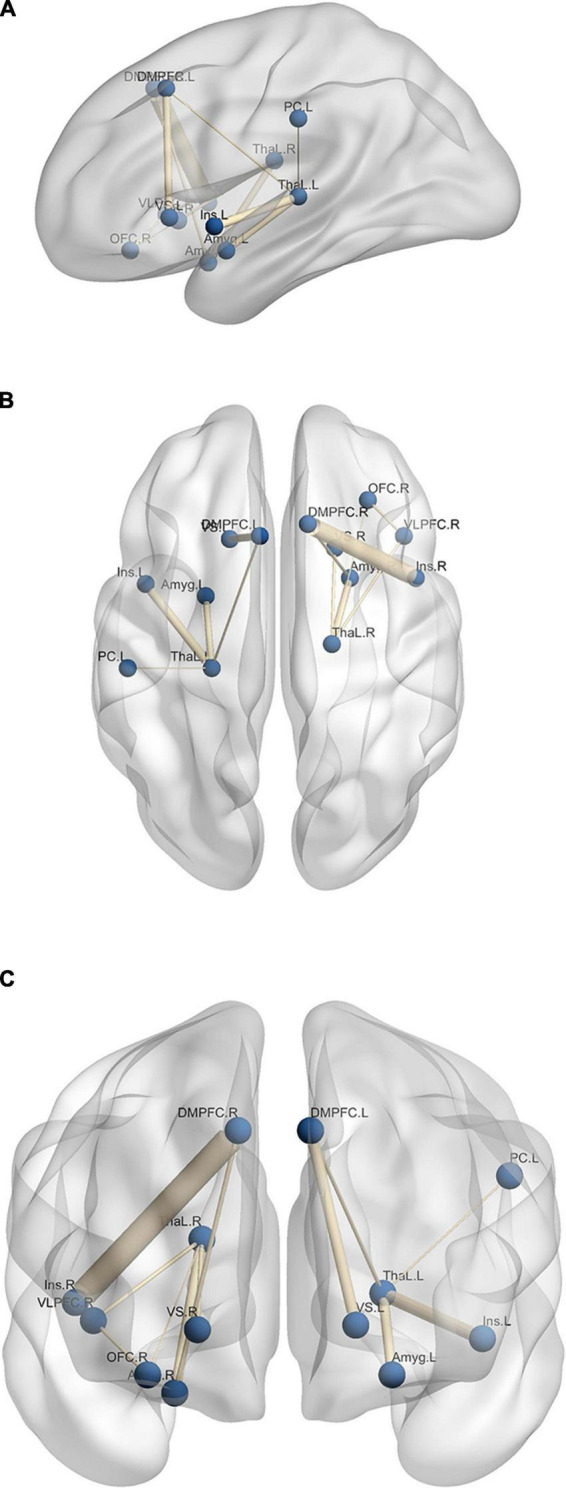
Significant edges of differential connectivity networks which show network links with significant changes between the Upregulation and View blocks for panel **(A)** sagittal, **(B)** axial, and **(C)** coronal layout. L, left; R, right; Amyg, amygdala; ThaL, thalamus; Ins, insula; OFC, orbitofrontal cortex; VS, ventral striatum; DMPFC, dorsomedial prefrontal cortex; VLPFC, ventrolateral prefrontal cortex; PC, postcentral.

The results of the experimental group in Figure 4 reveal the effect of neurofeedback during emotion regulation. To have a better understanding of the neural mechanisms of emotion regulation by the EEG neurofeedback and separate the effect of the neurofeedback and the recall of the autobiographical memories on Upregulation, the functional connectivity of the thirty-eight brain regions (including left/right amygdala, thalamus, insula, DMPFC, caudate, cuneus, hippocampus, posterior cingulate cortex, OFC, middle temporal gyrus, lingual gyrus, ventral striatum, DLPFC, ventrolateral prefrontal cortex (VLPFC), superior parietal, inferior parietal, supramarginal, postcentral and ACC are calculated during each block of the paradigm. Then, the significant connections between the Upregulation and View (Repeated Measures ANOVA for 38 × (38–1)/2 = 703 different connections; df = 179; FDR-corrected for multiple comparison; *q* = 0.05) for the experimental group are obtained. Functional connectivity is estimated by cross-correlation. Next, to reveal the effect of the neurofeedback, the significant links extracted from the experimental group are compared with those of the control group (two-sample *t*-test; df = 318; FDR-corrected for multiple comparison; *q* = 0.05). Connections significantly different between the two groups are due to the neurofeedback. Each illustrated edge in Figure 5 shows a connection with a significant change between the Upregulation and View blocks (and between the experimental and control groups) as a result of the neurofeedback. A thicker line between the regions corresponds to a higher correlation value.

Figure 5 illustrates the functional connectivity of the brain regions. Functional connectivity between several emotion-related regions increases in the Upregulation block, e.g., left amygdala and left thalamus. The roles of regions such as the amygdala, insula, thalamus, left ACC, hippocampus, OFC, and VLPFC in emotion regulation and recalling autobiographical happy memories are described in the Discussion Section.

**FIGURE 6:**
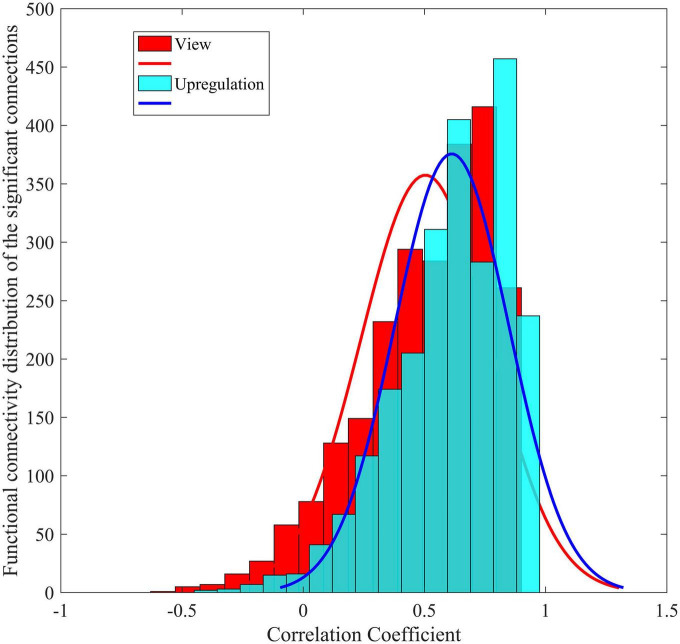
Functional connectivity distribution of Upregulation and View blocks for significant connections in the experimental group.

To demonstrate the distinction between the functional connectivity of the Upregulation and View blocks, the functional connectivity distribution of the significant connections in the experimental group is shown in Figure 6. The distributions of the Upregulation and View blocks show higher functional connectivity in the Upregulation blocks and therefore more synchronization among the brain regions during emotion regulation.

### Mood assessment

The emotional state test results obtained before and after the neurofeedback for the experimental and control groups are reported in Table 2. The results confirm the effectiveness of the neurofeedback in the experimental group. For the experimental group, the average scores for PANAS do not change significantly due to the neurofeedback but for the positive and negative mood states of PANAS, POMS, and Total Mood distribution (TMD), the changes are significant (paired *t*-test; *t*(17) = 4.01, p_positive mood states of PANAS_ = 4.5 × 10^–4^; *t*(17) = 3.32, p_negative mood states of PANAS_ = 2 × 10^–3^; *t*(17) = 3.61, p_*POMS*_ = 1.1 × 10^–3^; *t*(17) = 3.31, p_*TMD*_ = 2.1 × 10^–4^). This demonstrates that neurofeedback is effective in increasing the positive mood state and decreasing the negative mood state through recalling positive autobiographical memories. Changing the mean value of TMD from 7.5 to −4.7 may be interpreted as an increase in the positive mood and a decrease in the negative mood as a result of the neurofeedback.

**TABLE 2:** Psychometric test results before and after neurofeedback for the experimental and control groups.

Measure		Scores before neurofeedback	Scores after neurofeedback	Effect size (*d*)	T-score	*p*-value
PANAS	Experimental	52.2 ± 11.5	51.6 ± 8.8	-0.058	-0.54	0.7
	Control	54.2 ± 5.9	53 ± 4.8	-0.22	1.33	0.1
PANAS negative mood states	Experimental	20.8 ± 7.2	14.1 ± 4.8	-1.09	3.32	2 × 10^–3^
	Control	22.1 ± 6	19.3 ± 5	-0.50	1.6	0.064
PANAS positive mood states	Experimental	31.4 ± 6.1	37.5 ± 6.4	0.97	4.01	4.5 × 10^–4^
	Control	32.1 ± 5.7	33.7 ± 5.6	0.28	0.45	0.33
POMS	Experimental	24.6 ± 10.9	17 ± 6.9	-0.83	3.61	1.1 × 10^–3^
	Control group	27.1 ± 11.3	22.8 ± 11.1	-0.38	1.47	0.08
Total mood distribution (TMD)	Experimental	7.5 ± 11.5	–4.7 ± 7.1	-1.27	3.32	2.1 × 10^–4^
	Control	6.6 ± 11.2	3.6 ± 11.7	-0.26	1.07	0.15

For the control group, the scores of PANAS negative mood states show a change toward significance from before to after the neurofeedback (paired *t*-test; df = 13; *p*-value = 0.064) but for the positive mood states, they do not change significantly. This demonstrates that the recalling of the positive autobiographical memory even with sham neurofeedback is effective in decreasing (increasing) the negative (positive) mood state of the control group. The scores of POMS and Total Mood distribution (TMD) do not change significantly from before to after the neurofeedback. Changing the mean value of TMD from 6.6 to 3.6 for the control group may be interpreted as increasing the positive mood states and decreasing the negative mood states by recalling of the autobiographical memories even with sham neurofeedback. There are significant differences between the changes of the positive mood states of PANAS and TMD due to the neurofeedback in the experimental and control groups (two-sample *t*-test; df = 30; p_positive mood states of PANAS (Experimental group–Control group)_ = 0.02 and p_TMD (Experimental group–Control group)_ = 0.004).

Before and after the neurofeedback experiment, the Persian version of the Beck Anxiety Inventory (BAI) and State-Trait Anxiety Inventory (STAI) were completed by each participant to measure and evaluate the anxiety that might be induced by the MRI scanner. The results showed that the anxiety scores of all participants before and after the neurofeedback experiment were less than 16 and 33 for BAI and State Anxiety Inventory of STAI (in the range of minimal and mild anxiety), and after the neurofeedback test, the level of anxiety slightly decreased. Therefore, not only the anxiety did not increase during the experiment but also decreased as a result of the neurofeedback and emotion regulation. The mean ± standard deviation of the Beck Anxiety Inventory (BAI) and State Anxiety Inventory for “before neurofeedback” was 8 ± 4.4 and 27 ± 2.5 and for “after neurofeedback” were 6.6 ± 4.7 and 26.3 ± 2.8.

## Discussion

### A The role of activated brain regions in neurofeedback paradigm

In this study, the participants were able to upregulate positive emotion using the EEG neurofeedback based on the frontal asymmetry in the alpha frequency band and happiness induced task through retrieving their positive autobiographical memories. Comparison of the results of the experimental and control groups reveals the increased activity of the prefrontal, insular, and limbic regions and increased functional connectivity in/between the prefrontal, limbic, and insular regions because of the EEG neurofeedback in the experimental group. The psychometric tests confirm an increased positive emotion and a decreased negative emotion because of the neurofeedback. In our previous study (Zotev et al., 2013), we used the fMRI dataset used in this study for functional brain connectivity analysis. For this purposes, independent component analysis (ICA) was used to extract spatially independent functional networks of fMRI data without the need for prior model of the data or paradigm. Then, the temporal correlation between the time courses of these networks was calculated as functional network connectivity (FNC) of the independent component. Each network (IC) composed of several sub-regions and each region involved in different networks. ICA extracts and identifies networks rather than single voxels and each network composes of several regions. One of the weaknesses of ICA is that it is an exploratory data-analytic technique, and it provides no straightforward method of testing specific *a priori* hypotheses about any components. General linear model (GLM) is a model-based method to extract the activated brain voxels/regions according to the task model. Therefore, despite the ICA as a data driven method, GLM uses the model of the paradigm to extract activated brain voxels during the task. Therefore, in different study from our previous published study (Dehghani et al., 2020), first we quantified changes of EEG frontal asymmetry in the alpha frequency band during different blocks of experiment in both experimental and control groups. Then using the model of task and GLM, activated brain regions were extracted and functional connectivity between different activated brain regions in different blocks of experiments were calculated to extract the effect of neurofeedback. Finally, psychometric assessments before and after neurofeedback were compared to understand the effect of neurofeedback on emotion regulation.

As mentioned in the previous studies, several brain areas including the subcortical and limbic regions are involved in emotion regulation. Consistent with the previous studies, during emotion regulation, amygdala, insula, ACC, cuneus, caudate, OFC, VLPFC, ventral striatum, and temporal gyrus are activated (Table 1; Pelletier et al., 2003; Kim and Hamann, 2007; Burianova et al., 2010; Johnston et al., 2010, 2011; Ino et al., 2011; Bado et al., 2014; Zotev et al., 2014, 2016; Li et al., 2016; Lempert et al., 2017). As shown in Figure 5, the functional connectivity between several emotional-related regions changes during the Upregulation blocks relative to the View blocks.

According to the Table 3, comparison of the results of this study with those of previous studies on emotion regulation, revealed several new connectivity links including thalamus - DMPFC, thalamus – insula, thalamus - OFC, thalamus - VLPFC, thalamus – postcentral, DMPFC – ventral striatum, OFC - VLPFC. The extracted new connections can be justified according to emotion regulation models and role of those regions in emotion regulation and recalling positive autobiographical memories. For the functional connectivity analysis, in our previous study (Dehghani et al., 2020), ICA was used to extract the spatially independent functional networks. Then, the correlation between these networks was calculated as functional network connectivity (FNC) to extract the effect of neurofeedback. Using the ICA methods, two pairs of networks had significant connectivity differences between View and Upregulation blocks. Each network was composed of voxels from different regions, and each voxel was involved in several ICs, and the rationale behind using ICA was to see if there is any connection between independent networks without considering the task model. For the ICA analysis, the connectivity between network 9 (cuneus, precuneus, PCC, VLPFC, DLPFC) and network 22 (amygdala, caudate, hippocampus, insula, putamen, thalamus) and between network 26 (cuneus, middle occipital, fusiform, lingual gyrus) and network 44 (ventral striatum, VLPFC, DLPFC, OFC, inferior parietal, middle temporal gyrus) increased and most of connections are between occipital regions and limbic/parietal regions. However, the results of functional connectivity analysis using the activated brain regions by GLM revealed that increased connectivity are between limbic regions, limbic and prefrontal/parietal, and between prefrontal regions and the only common connectivity link between this study and our previous study is increased connectivity between thalamus and VLPFC.

**TABLE 3:** Significant connectivity obtained in previous emotion regulation researches and this study as a result of neurofeedback.

Study#	References	Brain area
1	Young et al., 2018	Amygdala and: inferior frontal G/lateral, medial PFC, medial frontopolar cortex, ventrolateral PFC, medial frontal Gyrus, ACC, insula, ventral striatum, putamen, thalamus, precuneus, cerebellum, temporal pole.
2	Paret et al., 2016b	Amygdala and: supplementary motor area, middle frontal gurus, brain stem, precuneus, ventromedial prefrontal cortex, white matter/right putamen/insula.
3	Zotev et al., 2011	Amygdala and : frontal lobe (ventrolateral prefrontal cortex, orbitofrontal cortex, middle frontal gyrus, superior frontal gyrus, ventromedial prefrontal cortex), temporal Lobe (middle temporal gyrus), limbic Lobe (hippocampus), sub-lobar Regions (insula, thalamus).
4	Paret et al., 2016a	Amygdala and: DLPFC, precentral gyrus, paracentral lobe, parahippocampal gyrus, extending to thalamus and hippocampus, cerebellum.
5	Herwig et al., 2019	Amygdala and: ACC, DLPFC, DMPFC, pre-SMA and VLPFC.
6	Koush et al., 2017	Amygdala-DLPFC.
7	Nicholson et al., 2017	Amygdala and: DMPFC, DLPFC, ACC.
8	Sarkheil et al., 2015	PFC-PCC.
9	Veit et al., 2012	Insula and: lingual gyrus, ventrolateral PFC, frontal inferior operculum, inferior orbitofrontal, middle frontal, middle orbitofrontal, occipital inferior, dorsal medial PFC.
10	Cohen Kadosh et al., 2016	Insula and: mid cingulate cortex, supplementary motor area, amygdala.
11	Ros et al., 2013	ACC and mid-cingulate cortex.
12	Banks et al., 2007	Amygdala and: OFC and DMPFC.
13	Morawetz et al., 2017	Inferior frontal gyrus (IFG) and: DLPFC, DMPFC, VMPFC, ACC, amygdala. Amygdala and prefrontal regions (e.g., DMPFC).
14	He et al., 2017	Amygdala and: ACC amd thalamus. PCC and centromedial amygdala (subregions of amygdala).
15	Koush et al., 2019	Temporoparietal junction (TPJ) and: SFG, DMPFC, vmPFC. vmPFC and: DMPFC, amygdala.
16	Dehghani et al., 2020	Occipital regions (cuneus, fusiform, lingual gyrus, middle occipital) and: Limbic system and sub-lobar (thalamus, hippocampus, amygdala, caudate, putamen, insula, ventral striatum), prefrontal and frontal cortex (VLPFC, DLPFC and OFC), parietal (inferior parietal), temporal (middle temporal gyrus). Parietal (precuneus) and: amygdala, hippocampus. PCC and: amygdala, hippocampus. VLPFC/DLPFC and: amygdala, insula, thalamus.
17	This study	Connections obtained in this study and reported in previous studies: amygdala – thalamus, amygdala - DMPFC, thalamus – ventral striatum, insula – DMPFC. New connections: thalamus – DMPFC, thalamus – insula, thalamus – OFC/VLPFC, thalamus – postcentral, DMPFC – ventral striatum, OFC – VLPFC.

^#^No, Numbers.

The limbic system and subcortical include several regions such as the hypothalamus, thalamus, amygdala, and hippocampus with key roles in emotion regulation. The amygdala plays a key role in emotion regulation/generation and connects with several emotion regulatory regions such as medial and lateral orbitofrontal cortices, ACC, and DLPFC through ventral and dorsal pathways (Phelps, 2004; Ochsner and Gross, 2008; Bracht et al., 2009). Thalamus activates a wide range of positive and negative emotional stimuli e.g., happiness, sadness, and disgust (Maddock et al., 2001; Zotev et al., 2018a). Thalamus is considered as a relay center for most of the sensory information. The sensory information first stops in the thalamus and then goes to destinations in the cortex (Cerqueira et al., 2008). The insula plays an important role in emotion processing and monitors internal emotional states e.g., disgust, happiness, and sadness (Chen et al., 2009; Pohl et al., 2013). Therefore, the increased activity of the thalamus, amygdala, insula and other regions e.g., the hippocampus, ACC, and ventral striatum is according to the role of these regions in emotion regulation and retrieving positive autobiographical memories (Bush et al., 2000; Hare et al., 2005; [Bibr B91][Bibr B98]; Suardi et al., 2016).

The lingual gyrus and cuneus from the occipital lobe are involved in visual processing and increased activity of these two regions was reported in previous studies of working memory, cognitive functions, visual processing, and memory retrieval (Gilboa et al., 2004; Burianova and Grady, 2007; Burianova et al., 2010; Vrticka et al., 2013; Deak et al., 2017). Therefore, according to the paradigm used in this study and visual processing of positive autobiographical memory images, the activity of these regions increased in this study.

Prefrontal/frontal cortex regions (OFC, VLPFC, and DLPFC) play key roles in emotion generation/regulation, self-monitoring, cognitive functions, working memory, recalling autobiographical memory, decision making, and pleasant emotional stimulus (Herrington et al., 2005; Beer et al., 2006; Schutter and van Honk, 2006; Svoboda et al., 2006; He et al., 2018). These regions have reciprocal connections with the other regions related to emotion regulation such as the amygdala and ACC. Therefore, the activity of the prefrontal/frontal cortex increased according to the role of these regions in emotion regulation, as described by several previous studies.

The increased activity of the parietal regions (superior parietal, inferior parietal, supramarginal, and postcentral) is related to the role of these regions in attention deployment to the presented images of positive autobiographical memories and their involvement in the integration of the sensory and behavioral information (Andersen, 1997; Bullier, 2001; Aday et al., 2017).

### B Network interpretation of functional connectivity analysis

According to the emotion regulation model described in Kohn et al. (2014), emotion regulation is modeled as a process with three steps. The first step is an appraisal of the stimulus. Subcortical regions such as the amygdala, basal ganglia, and ventral striatum play a key role in emotion generation. In this step, the affective arousal is relayed and projected to VLPFC *via* subcortical regions (amygdala, basal ganglia, and ventral striatum). The next step is “detecting and start of the regulation process” and the need for regulation is signaled by VLPFC and insula. The final step is regulation and change of the emotional state and moving to a new state. Thalamus directs the sensory information to different cortical and subcortical regions. Therefore, increasing the functional connectivity between amygdala and thalamus and between ventral striatum and thalamus are the results of passing visual stimulus information processed by the thalamus and occipital regions to the amygdala and ventral striatum for emotion generation and also the transmission of the somatosensory information as a result of emotion generation to the cortical regions by the thalamus. Decreased resting-state functional connectivity between the amygdala and thalamus was reported in a major depressive disorder study (Tang et al., 2018). Therefore, enhanced connectivity between the amygdala and thalamus is related to increased happiness and decreased sadness (based on psychometric assessment) as a result of emotion regulation and may be associated with decreased severity of the major depressive disorder.

The increased functional connectivity between thalamus and insula can be interpreted as the role of the insula in the second step of emotion regulation for sending the need for regulation to the cortical regions and its involvement in the third step of emotion regulation and the role of the thalamus as the relay center for sending sensory information to cortical and subcortical regions. The higher functional connection between the thalamus and insula was reported during meditation to reduce negative emotion (Jang et al., 2018) and seems to be similar to the effects of the neurofeedback paradigm used in this study. The increased functional connectivity between the thalamus and VLPFC may be interpreted as similar to those of the thalamus and insula. According to the proposed model in Kohn et al. (2014), VLPFC and insula have similar roles in emotion regulation. OFC receives input and sensory information from various brain regions such as the amygdala, hypothalamus, and insular cortex. Therefore, the rise of connectivity between OFC and thalamus originates from relaying sensory information by the thalamus and receiving it by OFC. There are indirect connections between the subcortical and prefrontal regions involved in emotion regulation. The functional connectivity between OFC and VLPFC, DMPFC and ventral striatum, DMPFC and amygdala, DMPFC and insula, thalamus and DMPFC are supported by extensive and reciprocal anatomical connections in the prefrontal cortex, between the prefrontal cortex and the limbic/paralimbic regions, and through indirect connection according to the steps of the emotion regulation models. The higher co-activation pattern between the limbic system (amygdala, insula, ventral striatum, and thalamus) and prefrontal and frontal cortex (DMPFC, OFC, and VLPFC) was associated with the effectiveness of emotion regulation and less intensity of negative affect and lower level of anxiety, especially lower functional connectivity was reported between thalamus/caudate and OFC among major depressive participants (Banks et al., 2007; Lui et al., 2011; Cheng et al., 2016; Lithari et al., 2016; Kong et al., 2018).

The increased functional connectivity between the thalamus and postcentral (as a part of the parietal lobe) is related to the role of the parietal to receive and process sensory information (e.g., visual information) from the thalamus as a relay center for sending sensory information.

The functional connectivity results of this study are consistent with the previous emotion regulation studies and models. There are some new connections identified in this study that are not mentioned in the previous studies. The new connections are between the thalamus and DMPFC, thalamus and insula, thalamus and OFC, thalamus and VLPFC, thalamus and postcentral (parietal), DMPFC and ventral striatum, and VLPFC and OFC. In Table 3, the connectivity identified in this study and those of the previous emotion regulation studies (using different strategies and not limited to neurofeedback and recalling autobiographical memories) is presented. We reported them because of the concept of emotion regulation used in those studies and the existence of some brain circuits activated regardless of the type of stimulus. We also wanted to illustrate that some of the connections identified in this study were not reported in the previous emotion regulation studies (even with different paradigms).

### C Limitations

Due to the high cost, high time consumption, and practical limitation in the simultaneous recording of EEG and fMRI, e.g., installation of EEG electrodes, the sample sizes of such studies are small (Kinreich et al., 2012; Zotev et al., 2014, 2016; Zich et al., 2015; Perronnet et al., 2017; Cury et al., 2020; Dehghani et al., 2020; Lioi et al., 2020b; Ebrahimzadeh et al., 2021; Ogawa et al., 2021). However, comparing the number of participants in this study (*n* = 32) with those in a recent literature review on emotion regulation-based fMRI neurofeedback (Linhartová et al., 2019), our study has a sample size similar to or higher than 50 of the 55 studies reviewed. A limitation of this study was using only males. It was difficult to enroll females in this study. Therefore, generalization of the results is difficult based on the previous reports of gender-specific differences in emotion regulation (McRae et al., 2008; Mak et al., 2009a,b; Whittle et al., 2017). Future studies can be done on the female subjects using the same emotion regulation paradigm.

## Conclusion

This study demonstrates the effectiveness of the EEG neurofeedback in changing EEG and fMRI signals. It also shows changes in the functional connectivity of the brain regions involved in emotion regulation, especially between the frontal, parietal, limbic, and insular regions with some new connectivity links not mentioned in previous studies. The EEG neurofeedback using the proposed happy autobiographical memory paradigm changes the fMRI BOLD signal of various brain regions and the EEG frontal asymmetry more than those observed in the previous studies, even when they used both modalities for neurofeedback (Johnston et al., 2010; Young et al., 2014; Zotev et al., 2014, 2016; Li et al., 2016). Comparison of the psychometric test results obtained before and after the neurofeedback experiment, confirms the effectiveness of the neurofeedback paradigm in changing the negative and positive mood states of the participants.

The increased functional connectivity between prefrontal/frontal and other brain regions may provide valuable information, particularly in the EEG neurofeedback studies, where the prefrontal activity can be measured by EEG and modulation/activity of other regions can be predicted. The results may suggest the use of the proposed paradigm for the treatment of mental disorders in a small number of treatment sessions because of the larger changes and effect size in the EEG and fMRI signals and the psychometric assessments.

The proposed connectivity analysis may be used in neurofeedback studies in addition to the powers of EEG and the fMRI BOLD signals. The brain is a complex network, and the function of each region affects the others. In most brain functions and mental disorders, multiple brain regions are involved. Therefore, using feedback based on the connectivity of the involved regions for changing the whole network for a specific paradigm or mental disorder may be more effective than the alternative methods and would be an avenue of further investigation (Sulzer et al., 2013; Linhartová et al., 2019).

## Data availability statement

The raw data supporting the conclusions of this article will be made available by the authors, without undue reservation.

## Ethics statement

The studies involving human participants were reviewed and approved by the Iran University of Medical Sciences, Tehran, Iran. The patients/participants provided their written informed consent to participate in this study.

## Author contributions

All authors listed have made a substantial, direct, and intellectual contribution to the work, and approved it for publication.
