# Supplementary material for: Probing fMRI brain connectivity and activity changes during emotion regulation by EEG neurofeedback
Source: Front Hum Neurosci. 2023 Jan 6;16:988890. doi: 10.3389/fnhum.2022.988890 (PMC9853008; doi:10.3389/fnhum.2022.988890)
Supplement: Supplementary file 1 [file Data_Sheet_1.PDF]

Supplementary materials

1- Real-time and offline values of neurofeedback

For high-quality neurofeedback, the following steps were done. First, the power spectrum of the data recorded inside and outside of the MRI scanner was compared to evaluate the quality of the denoised data. Secondly, the spectrum of neurofeedback and its values obtained by RecView were compared with the results of the offline analysis to evaluate the neurofeedback quality. The results confirmed the quality of the offline removal of the EEG artifacts and the reliability of the neurofeedback. They also proved that the neurofeedback provided to each participant was based on the actual brain activity, not the artifacts. For this purpose, the following figure shows the real-time values of neurofeedback using real-time denoised EEG signal by Recview software and offline analysis of EEG data using FMRIB plug-in as a Matlab toolbox and other steps e.g., low-pass filtering and ICA analysis. More details of the offline analysis were described in the manuscript.

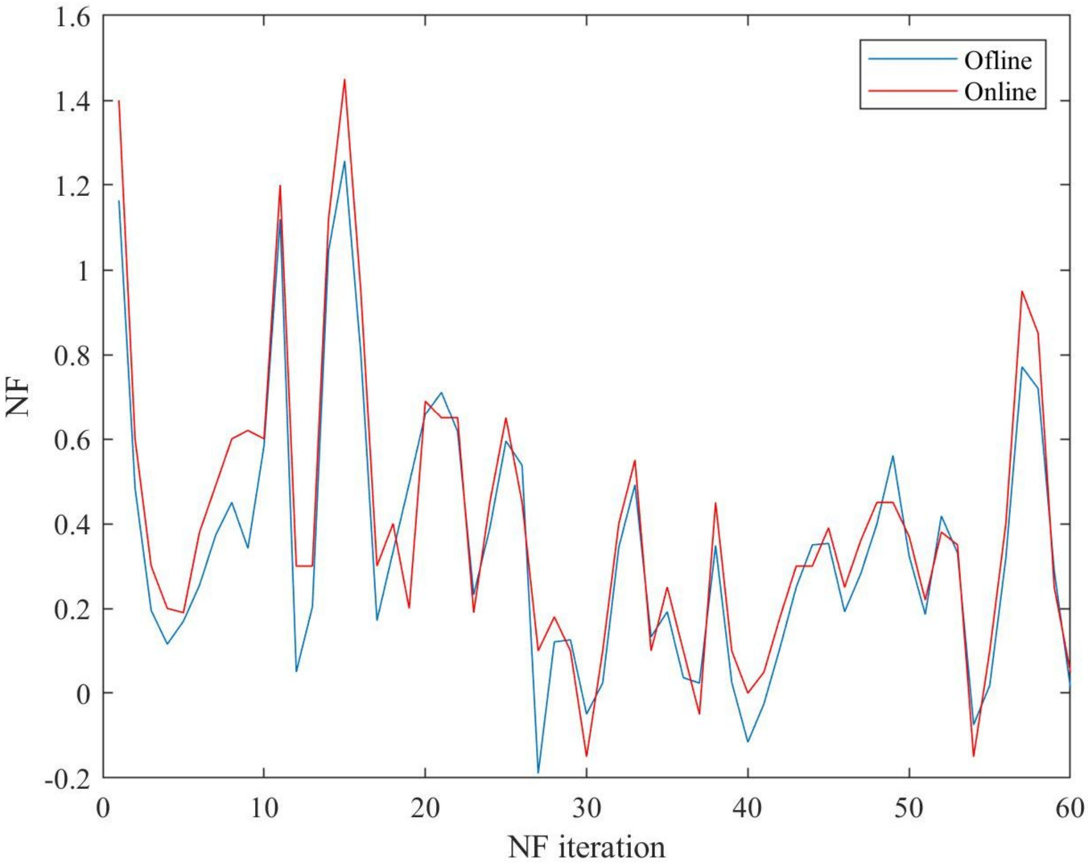

Fig1: Comparison of the NF values for online and offline analysis averaged across all participants

2- Activation map for Upregulation versus View in the experimental group

Pre-processing of a single-subject fMRI data includes slice-timing correction, motion correction, temporal high pass filtering (cut-off = 0.005 Hz), and spatially smoothing using an 8 mm full-width at half-maximum Gaussian kernel. The standard GLM analysis is then applied to the fMRI time series. Three regressors for Upregulation, View, and Rest are convolved with the hemodynamic response function and six motion confounds are included in the GLM model. Finally, the whole brain is thresholded at p-value = 0.01 for voxels and for cluster correction at p-value = 0.01 in the cluster-level correction algorithm, which corrects for the multiple comparisons using the Gaussian Random Field (GRF) model. For better understanding, the following figure presents the activated brains of the amygdala.

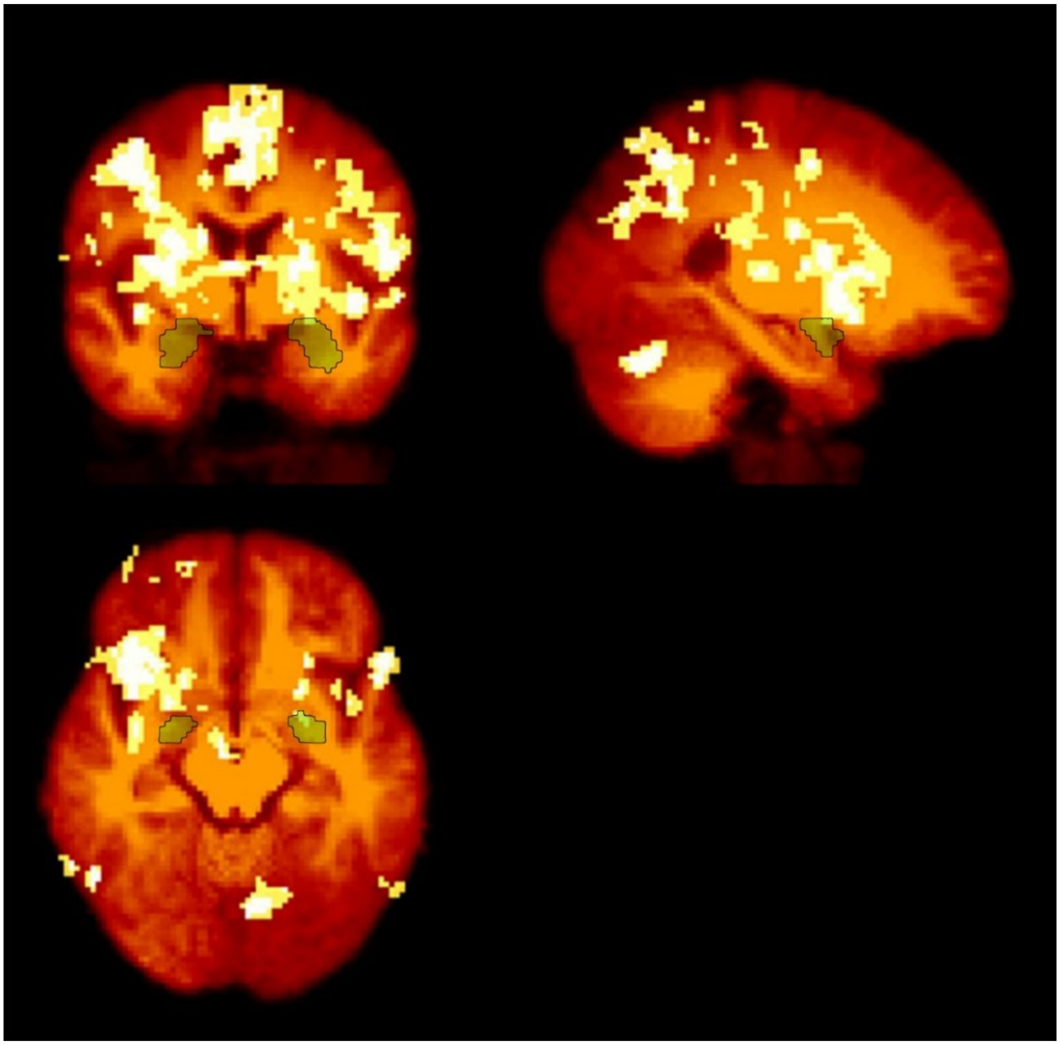

Fig2: Activated brain regions masked by amygdala with CARDIAC color.

3- Positive autobiographical memories

Before the experiment, each participant was interviewed and asked to write several positive autobiographical memories. Samples of the positive autobiographical memories written by the subjects are as following:

- Winning the match/game
- Wedding or marriage ceremony
- Admire, support, and encourage by others
- Party
- Meeting (friends, family, etc.)
- Trip to …
- Working at …
- First day of employment
- Birth of son/daughter, nephew, …
- Holiday in …
- Memories with the first friend
- Going to cinema or movie with friends
- Watching the football match between X and Y in the stadium
- First day of university
- Admitted to university
- Concert
- Exercise
- Visiting a special place
- Barbecue with family/friends
- Helping ….
- Listening to music
- Driving a car in the Chaloos road
- Buying the first car/house

4- BOLD signal change for control group

The changes of the BOLD signal of brain regions in Table 1 for the Upregulation versus View and Rest in the experimental group are significantly different from those of the control group. The signal changes for the participants in the control group are listed in the Table 1, for most regions, the signal changes were less than 0.20%.

Table 1: signal changes for the participants in the experimental and control groups.

Regions	Experimental group		Control group	
	Sig % UP-View	Sig % Up-Rest	Sig % UP-View	Sig % Up-Rest
Left Amygdala	0.86	0.70	0.15	0.2
Right Amygdala	0.65	0.72	0.18	0.15
Left Insula	1	0.64	0.1	0.2
Right Insula	0.91	0.62	0.12	0.1
Left Anterior Cingulate Cortex	0.97	0.81	0.20	0.25
Right Anterior Cingulate Cortex	0.64	0.38	0.11	0.16
Left Cuneus	0.45	1.56	0.12	0.25
Right Cuneus	0.40	1.90	0.08	0.18
Left Lingual Gyrus	1.21	1.39	0.2	0.25
Left Posterior Cingulate Cortex	0.49	0.33	0.1	0.05
Left Thalamus	1.07	0.85	0.2	0.18
Right Thalamus	0.86	0.65	0.1	0.15
Left Caudate	0.86	0.65	0.18	0.2
Right Caudate	0.74	0.49	0.1	0.15
Left Hippocampus	0.57	0.56	0.05	0.1
Right Hippocampus	0.44	0.59	0.1	0.15
Left Dorsomedial Prefrontal Cortex	0.85	1.02	0.1	0.08
Right Dorsomedial Prefrontal Cortex	0.37	0.81	0.05	0.13
Left Orbitofrontal Cortex	1.13	1.04	0.2	0.18
Right Orbitofrontal Cortex	1.12	0.81	0.2	0.25
Left Middle Temporal Gyrus	0.66	0.59	0.1	0.12
Right Middle Temporal Gyrus	0.69	0.70	0.1	0.12
Left Ventral Striatum	1.17	0.84	0.15	0.1
Right Ventral Striatum	0.81	0.66	0.15	0.1
Left Ventrolateral Prefrontal Cortex	0.67	0.81	0.1	0.15
Right Ventrolateral Prefrontal Cortex	0.65	0.58	0.1	0.05
Left Dorsolateral Prefrontal Cortex	0.84	0.90	0.1	0.15
Right Dorsolateral Prefrontal Cortex	0.76	0.75	0.12	0.15
Left Superior Parietal	0.46	0.69	0.05	0.1
Right Superior Parietal	0.33	0.89	0.04	0.15
Left Inferior Parietal	0.60	0.41	0.14	0.15
